# Relationship between Exercise Capacity and Brain Size in Mammals

**DOI:** 10.1371/journal.pone.0020601

**Published:** 2011-06-22

**Authors:** David A. Raichlen, Adam D. Gordon

**Affiliations:** 1 School of Anthropology, University of Arizona, Tucson, Arizona, United States of America; 2 Department of Anthropology, University at Albany–SUNY, Albany, New York, United States of America; University of Lethbridge, Canada

## Abstract

**Background:**

A great deal of experimental research supports strong associations between exercise, cognition, neurogenesis and neuroprotection in mammals. Much of this work has focused on neurogenesis in individual subjects in a limited number of species. However, no study to date has examined the relationship between exercise and neurobiology across a wide range of mammalian taxa. It is possible that exercise and neurobiology are related across evolutionary time. To test this hypothesis, this study examines the association between exercise and brain size across a wide range of mammals.

**Methodology/Principal Findings:**

Controlling for associations with body size, we examined the correlation between brain size and a proxy for exercise frequency and capacity, maximum metabolic rate (MMR; ml O_2_ min^−1^). We collected brain sizes and MMRs from the literature and calculated residuals from the least-squares regression line describing the relationship between body mass and each variable of interest. We then analyzed the correlation between residual brain size and residual MMR both before and after controlling for phylogeny using phylogenetic independent contrasts. We found a significant positive correlation between maximum metabolic rate and brain size across a wide range of taxa.

**Conclusions:**

These results suggest a novel hypothesis that links brain size to the evolution of locomotor behaviors in a wide variety of mammalian species. In the end, we suggest that some portion of brain size in nonhuman mammals may have evolved in conjunction with increases in exercise capacity rather than solely in response to selection related to cognitive abilities.

## Introduction

A large amount of recent research has detailed associations between exercise, neurogenesis, cognition, and the size of certain brain structures in both humans and nonhuman animal models [1]–[6]. Aerobic exercise increases levels of compounds such as brain derived neurotrophic factor (BDNF), insulin-like growth factor 1 (IGF-1), and vascular endothelial growth factor (VEGF) [7], which appear to lead to exercise-induced neurogenesis in the rodent and human hippocampus [5]. Additionally, Chapell et al. [8] showed that intra-specific variation in maximum voluntary metabolic rate is positively correlated with *total* brain mass in gerbils, suggesting that exercise may affect structures outside of the hippocampus, at least in some species. While these studies point to clear links between neurobiology and exercise within individuals and within a species, to date, no study has examined the relationship between exercise and neurobiology interspecifically. Here, we test the hypothesis that exercise and neurobiology are related across a broad range of mammalian species.

Testing this hypothesis requires comparative measures of both neurobiology and exercise capacity. Among mammals, the most extensive neurobiological data available are overall brain sizes. The size of the brain, relative to body mass, is often considered a determining factor in cognitive abilities [9]–[11], behavioral flexibility [12], and intelligence [13]–[15]. Examining the association between exercise and total brain size is therefore ecologically and evolutionarily relevant. However, there are limitations to examining overall brain size, rather than the evolution of specific brain components [16]. Increases in brain size are the result of changes in the size of different brain components, and examinations of total brain size do not take into account the fact that selection on brain components may differ substantially [16], [17]. Despite this downside to the use of total brain size, it remains the only neurobiological variable for which we have a substantial mammalian database. Additionally, overall brain size may be more appropriate in this study, since we are not concerned with correlations between specific cognitive functions and behavior, but with a more general correlation between physical activity and neurobiology.

In addition to a measure of neurobiology, we require an index of athleticism and exercise frequency across a wide range of mammals. We use maximal metabolic rate (MMR; ml O_2_ min^−1^) as a proxy for exercise frequency or athleticism. MMR is the maximum level of oxygen consumed by an individual during exercise before anaerobic metabolism begins supplying energy [18]. Because MMR sets the upper aerobic limit for exercise in organisms, it is related to an individual's capacity for aerobically supported activity and overall aerobic scope [19], [20], and is correlated with an individual's total amount of sub-maximal aerobic activity [21], [22]. Thus, relatively high MMRs in mammals are likely tied to relatively high levels of aerobic activity. Based on the relationship between MMR and exercise levels, we predict that relatively high MMRs are associated with relatively large brain sizes. Support for this prediction would link neurobiology and exercise capacity across mammals for the first time.

## Methods

Brain masses and MMRs were collected for 29 mammalian species from data compiled by Isler and van Schaik [23] and Weibel et al. [18] respectively (Table 1). The sample size is necessarily small given the difficulty of collecting MMR data that are reliable across taxa. Data selected came from studies where animals ran on a treadmill at varying intensity, changing speed and/or incline, and where oxygen consumption and plasma lactate concentrations were measured [18]. For each run in these studies, speed was held constant, and was varied only between runs [18]. MMR was determined as the level of oxygen consumption when a further increase in work output (e.g., speed) does not cause a change in oxygen consumption, but does cause a change in plasma lactate concentrations, since the additional energy required for the increased work output comes from anaerobic metabolism [18]. We excluded the calf and pony from Weibel et al. [18] to maintain species independence in the data set (the data set includes adult cattle and horses).

**Table 1:** Brain mass and MMR data.

Genus	Species	Body Mass (g)	VO_2_ Max (ml/min)	Body Mass (g)	Brain mass (g)	References
*Alopex*	*lagopus*	4510	897.50	3415.00	35.52	[55], [56]
*Antilocapra*	*americana*	28400	8435.00	35369.16	145.78	[51], [57], [58]
*Apodemus*	*sylvaticus*	20	5.28	22.65	0.58	[50], [59]
*Bettongia*	*penicillata*	1100	194.70	981.00	9.56	[60]
***Bos***	***taurus***	475000	24225.00	490500.00	454.40	[61]–[63]
***Canis***	***familiaris***	25900	3825.00	14560.00	79.99	[64]
*Canis*	*latrans*	12400	2283.30	9135.00	83.37	[55], [59], [64]
*Canis*	*lupus*	27600	4310.00	28700.00	132.75	[55], [65]
***Capra***	***hircus***	24300	1344.70	28830.00	110.50	[62], [64]
***Cavia***	***porcellus***	584	21.49	835.50	4.51	[62], [66]
*Connochaetes*	*taurinus*	102000	4468.00	156577.99	364.33	[51], [67], [68]
***Equus***	***caballlus***	453000	56005.00	412360.93	702.50	[69]
*Gazella*	*granti*	10100	539.30	49022.00	148.74	[51], [58], [70]
*Genetta*	*tigrina*	1380	146.60	1750.00	15.18	[55], [59]
*Helogale*	*parvula*	430	32.59	267.00	4.76	[55]
*Homo*	*sapiens*	77940	5694.00	60838.90	1311.40	[64]
*Kobus*	*defassa*	110000	5172.00	229643.38	314.61	[51], [67], [68]
*Madoqua*	*kirkii*	4200	228.10	4457.94	34.31	[51], [64], [71]
*Mungos*	*mungo*	1140	130.00	860.00	10.49	[55], [59]
*Mus*	*musculus*	26	3.88	19.55	0.44	[50], [62]
*Neotragus*	*moschatus*	3300	317.80	3292.97	33.18	[51], [72]
***Ovis***	***aries***	21800	1013.70	51900.00	132.50	[62], [73]
*Panthera*	*leo*	30000	1800.00	157400.00	238.50	[55], [59], [64]
*Peromyscus*	*maniculatus*	22	4.93	20.13	0.63	[59], [62], [74]
***Rattus***	***norvegicus***	278	54.44	290.29	2.31	[65], [75]
*Spalax*	*ehrenbergi*	136	23.13	146.60	1.88	[50], [76]
***Sus***	***scrofa***	18500	1731.60	132000.00	186.60	[62], [77]–[79]
*Tamias*	*striatus*	90	14.58	93.00	2.20	[50], [59], [64]
*Taurotragus*	*oryx*	240000	8640.00	480000.00	460.00	[62], [67]

Notes: The majority of brain mass data were compiled by Isler and van Schaik [23]. All MMR data were compiled by Weibel et al. [18]. Data from Mace et al. [50] are corrected values following Isler and van Schaik [23]. Species in bold were not included in the wild-only analyses.

Brain masses and associated body masses were collected largely from the carefully compiled database in Isler and van Schaik [23]. Because human brain size relative to body size is greater than six standard deviations higher than the other mammals included in this study (see below), we performed an analysis first on nonhuman mammals, and then performed a second analysis including humans. For both analyses (including and excluding humans), we calculated residuals from ordinary least-squares (OLS) regression lines describing the relationships between body mass and MMR and body mass and brain size in logged space. We then calculated the Pearson-moment correlation between residuals of brain size from body mass and residuals of MMR from body mass.

**Figure 1:**
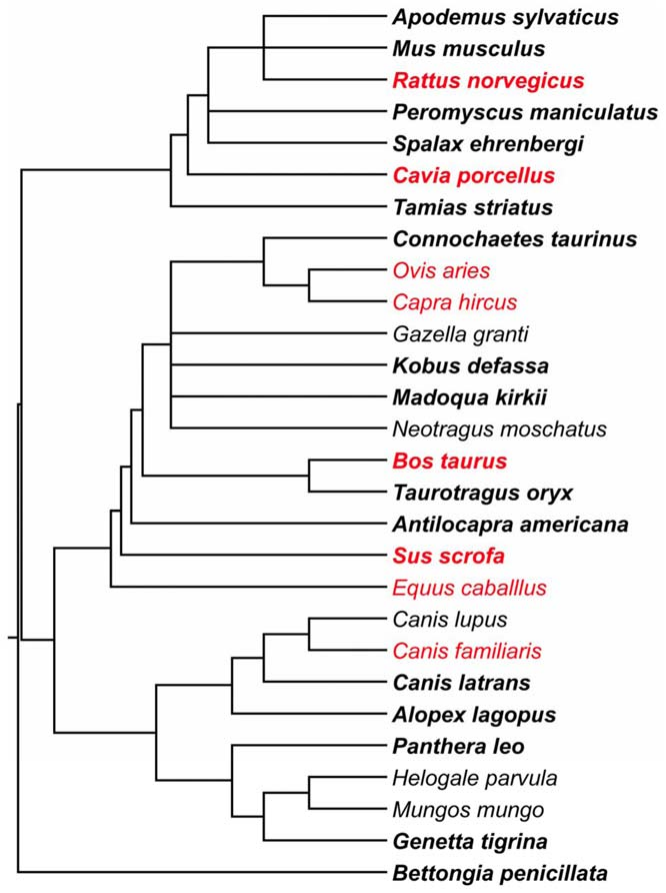
Phylogeny used in this analysis. Taxa which are not included in the wild sample are shown in red. Taxa in bold have BMR data in addition to MMR data. Branch lengths shown here were assigned following Nee [27].

Phylogenetically independent contrasts (PIC) were calculated for MMR, brain mass, body mass associated with the MMR data set, and body mass associated with the brain mass data set using the PDAP:PDTree module version 1.15 in Mesquite version 2.73 [24], [25]. The phylogenetic branching sequence is taken from Bininda-Emonds et al. [26] (see Fig. 1). Absolute values of standardized contrasts using estimated divergence times were negatively correlated with contrast standard deviations, so branch lengths were assigned using the method of Nee [27] where the distance from the tips to a given node is equal to the logarithm of the number of tips descending from that node. Absolute values of the resulting contrasts were uncorrelated with contrast standard deviations as suggested by Garland et al. [28]. Contrasts residuals were calculated from least-squares regressions against body mass contrasts where the intercept was constrained to equal zero.

## Results

**Figure 2:**
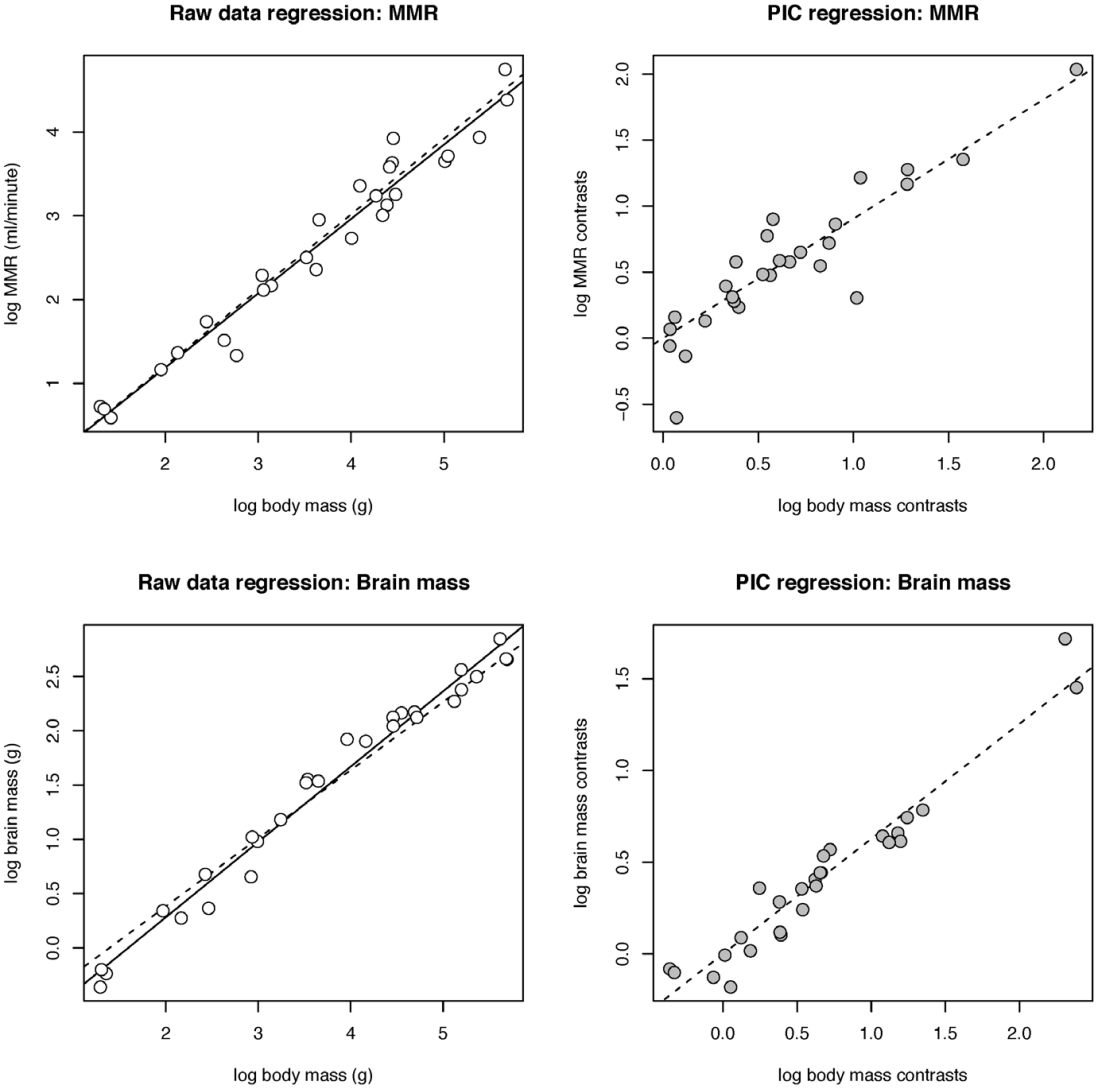
Relationships between body size, brain size and exercise capacity. A) and B) Relationship between body mass and MMR before and after controlling for phylogeny using PIC. C) and D) Relationship between body mass and brain size before and after controlling for phylogeny using PIC. Ordinary least squares regression lines shown as solid lines for raw data and as dotted lines for PIC. PIC regressions have been superimposed on raw data by passing them through the phylogenetically-weighted grand mean of the data sets following Garland and Ives [54].

**Figure 3:**
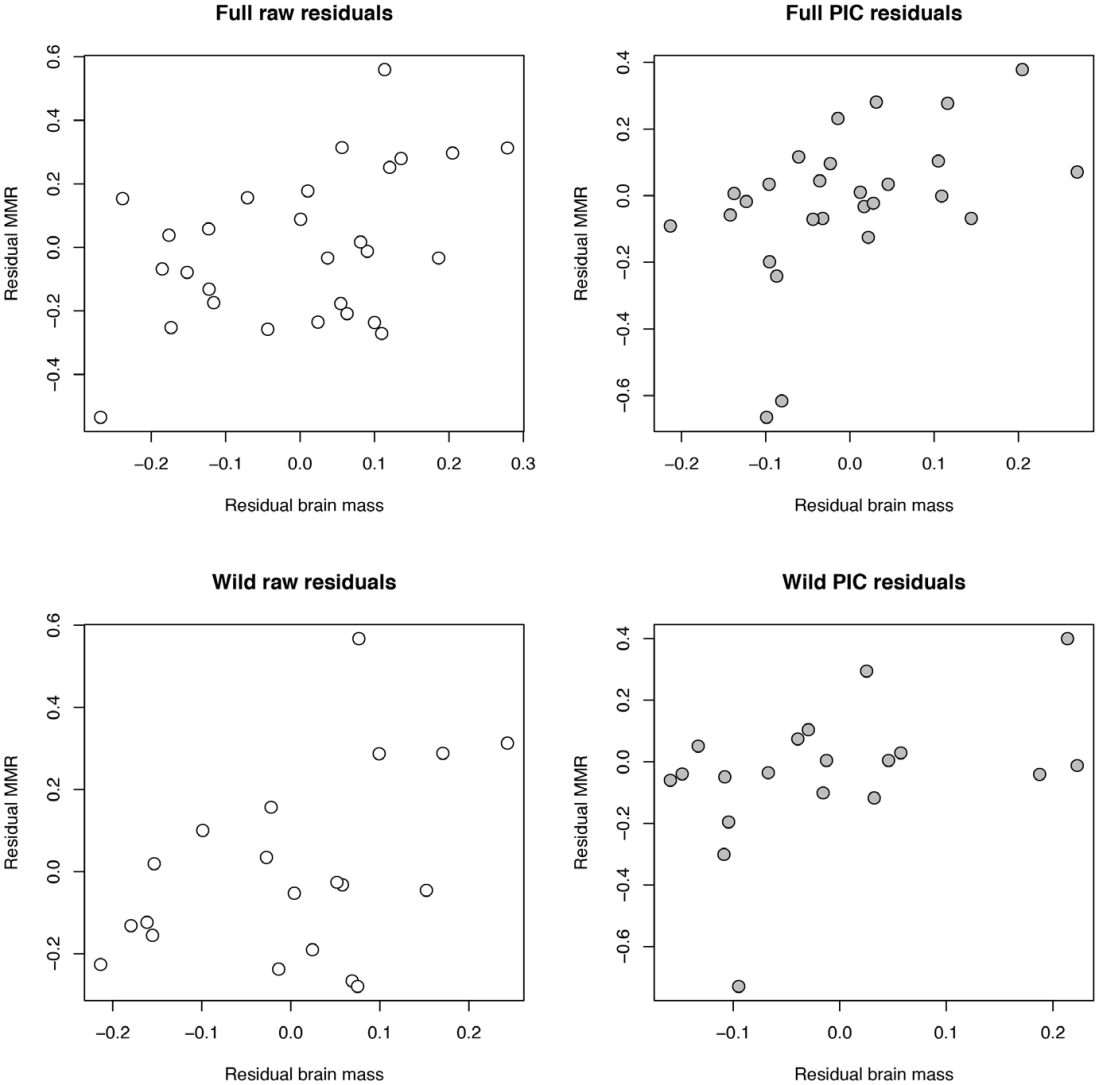
Relationship between raw and independent contrasts of residuals of brain mass from body mass and residuals of MMR from body mass in the full sample (A and B respectively) and the wild-only sample (C and D respectively).

Both brain size and MMR are significantly correlated with body mass before and after controlling for phylogeny (Fig. 2). After removing the effects of body mass using a residuals analysis, we found that variation in brain mass across a wide range of nonhuman mammals is significantly positively correlated with variation in MMR (Fig. 3a; Table 2). This relationship remains significant after accounting for phylogeny using independent contrasts (Fig. 3b), and after removing domesticated species from the analysis (Fig. 3c; Table 2). The PIC results for the wild-only sample (Fig. 3d) border on significance with p = 0.069. In addition, the relationship between residual MMR and residual brain size is not due to an overall correlation between basal metabolism and brain size in this sample, as residual BMR and residual brain size are not significantly correlated with each other (Table 2 and Table 3). When humans are included, the relationship between MMR and brain size residuals is no longer significant in analyses of both raw data and PIC (r_raw_ = 0.29, p_raw_ = 0.13; r_PIC_ = 0.21, p_PIC_ = 0.29), due primarily to the strong leverage of the human brain mass residual in the analysis of the raw data, and of the residual brain mass contrast between humans and Rodentia in the PIC analysis. Using equations for the full raw (non-phylogenetically controlled) data sample to calculate residuals of the humans from the mammalian regression lines, human MMR is slightly larger than that expected for their body size (residual is 0.03 standard deviations greater than expected), while human brain size is considerably larger than expected (residual is 6.36 standard deviations greater than expected).

**Table 2:** Relationship between residual brain mass and residual metabolic rate.

Metabolic variable	Method	Sample	N	r	p
MMR	Raw	Full	28	0.440	0.019
MMR	Raw	Wild	20	0.447	0.048
MMR	PIC	Full	27	0.459	0.016
MMR	PIC	Wild	19	0.427	0.069
BMR	Raw	Full	19	0.155	0.525
BMR	Raw	Wild	15	0.362	0.186
BMR	PIC	Full	18	0.184	0.462
BMR	PIC	Wild	14	0.383	0.176

Full sample refers to all taxa except *Homo sapiens*; domesticated taxa have been removed to produce the wild sample.

**Table 3:** Basal metabolic rates.

Genus	Species	BM (g)	BMR (W)
*Alopex*	*lagopus*	3600.00	7.67
*Antilocapra*	*americana*	34799.30	50.97
*Apodemus*	*sylvaticus*	23.80	0.26
*Bettongia*	*penicillata*	1018.00	3.13
*Bos*	*taurus*	347000.00	306.77
*Canis*	*latrans*	10148.90	19.42
*Cavia*	*porcellus*	639.10	2.13
*Connochaetes*	*taurinus*	196500.00	230.07
*Genetta*	*tigrina*	1699.00	4.19
*Kobus*	*defassa*	100000.00	148.95
*Madoqua*	*kirkii*	4290.00	11.97
*Mus*	*musculus*	18.00	0.27
*Panthera*	*leo*	98000.00	94580.00
*Peromyscus*	*maniculatus*	20.50	0.22
*Rattus*	*norvegicus*	206.90	1.40
*Spalax*	*ehrenbergi*	133.80	0.59
*Sus*	*scrofa*	135000.00	104.15
*Tamias*	*striatus*	89.60	0.81
*Taurotragus*	*oryx*	141403.70	190.21

Note: All BMRs from Savage et al. [80].

## Discussion

These results suggest a positive association between exercise capacity and brain size in nonhuman mammals. Our results are consistent with recent intra-specific data that also show a relationship between exercise and brain size. For example, Chappell et al. [8] showed that total brain size is positively correlated with maximum voluntary aerobic capacity (metabolic rate at the maximum voluntary wheel running speed) in gerbils. Another recent study focused on mice that were part of a selection experiment for high amounts of voluntary wheel running [29]. Mice from selected lines run significantly farther than mice from control lines when given voluntary access to running wheels and have higher VO_2,max_ compared to control lines [30]. Preliminary data show that brain components such as the mid-brain and the dentate gyrus were significantly larger in selected mice compared to control mice [30]–[32]. Combined with the results of our study, we now have strong evidence that increased exercise capacity, both intra- and inter-specifically, is correlated with increased brain size and there is a suggestion from selection experiments that this relationship can arise from the evolution of increased exercise frequency.

However, the mechanisms responsible for the relationship between total brain size and exercise capacity remain unclear. The correlation identified in this study may indicate an increase in brain size in response to increased exercise capacity, an increase in exercise capacity associated with metabolic changes required to support increased brain size, or a response in both relative brain size and exercise capacity to some third factor. Comparative studies such as this one cannot easily identify causal mechanisms behind correlations, however they should lead to experimental research that can identify such mechanisms [16]. Below, we present one possible explanation for the association between brain size variation and variation in exercise capacity across mammals. Although necessarily speculative in nature, this hypothesis is based on a large body of experimental data and lays the foundation for future studies.

### Possible mechanism

One mechanism that could explain the relationship between exercise and brain size in mammals relates known causes of exercise-induced adult neurogenesis to fetal and postnatal neurodevelopment. It is important to note that mechanisms that explain the results from this study must only account for a small amount of brain size variation among mammals (i.e., Table 2). Thus, slight alterations in total brain growth, or the growth of specific brain structures, could lead to the association between exercise and brain size found across mammalian taxa.

Experimental work has shown that the adult brain is plastic and that exercise can increase neurogenesis in the hippocampus, primarily through the upregulation of neurotrophins and growth factors [3], [5]. For example, voluntary running in rodents results in a significant upregulation of brain-derived neurotrophic factor (BDNF), which improves neuronal survival and leads to neurogenesis [7], [33]. Additionally, circulating concentrations of insulin-like growth factor 1 (IGF-1) and vascular endothelial growth factor (VEGF) also increase with exercise [34], [35]. These growth factors are likely produced peripherally to aid glucose metabolism and for angiogenesis related to exercise performance (e.g., repair and growth of blood vessels to support skeletal muscle) [3], [36], [37]. These compounds then readily pass through the blood-brain barrier and interact with BDNF to lead to neurogenesis and neuroprotection [3].

The same compounds that influence adult neurogenesis (i.e., increased circulating levels of BDNF, IGF-1, and VEGF) profoundly affect brain growth and development [38], [39]. Experimental work in rodents has shown that deficiencies in these growth factors or neurotrophins during development lead to smaller overall adult brain sizes [38], while overexpression leads to significantly larger brain sizes at adulthood [39]. In humans, low serum levels of growth factors are correlated with reduced postnatal brain growth [40]. Thus, the interspecific association between brain size and exercise may be due to an increase in circulating neurotrophins and growth factors in mammals with increased exercise capacities.

Recent work does suggest that increased exercise capacity is associated with higher overall levels of neurotrophins and growth factors in mammals. For example, variation in exercise capacity in different rat strains is associated with variation in BDNF levels *at rest* [41]. Rats from strains that voluntarily run long distances have significantly higher levels of BDNF at rest than rats from strains that do not run as far [41]. Importantly, BDNF levels were measured in rats that were not given access to running wheels, suggesting the differences in BDNF levels are not simply plastic responses to exercise during their lifetimes, but rather reflect differences between strains accumulated over multiple generations [41]. Additionally, mice that were part of the selection experiment described above (i.e., selected for high amounts of voluntary wheel-running [29]) evolved higher MMRs than control lines [42], and had higher BDNF levels following several days of voluntary wheel-running compared to control lines [43]. At rest, selected mouse lines also had significantly higher levels of circulating VEGF compared to control lines [44]. Since BDNF and VEGF are known to play a major role in early neurodevelopment [39], [45], the evolution of high amounts of voluntary exercise increases circulating levels of compounds essential for the development of the brain.

There is also circumstantial evidence that circulating VEGF levels may be greater across mammals with relatively higher MMRs. There is a strong correlation across mammals between MMR and the volume of the muscle capillary network, which is well explained by the need to deliver oxygen to working muscles during aerobic exercise [18]. Several lines of evidence indicate that skeletal muscle capillaries are regulated by VEGF [46], suggesting an overall relationship between VEGF and MMR. For example, if endogenous VEGF production is inhibited, skeletal muscle capillarization is greatly reduced (by up to 64%) [47]. Additionally, blockage of VEGF during exercise results in inhibition of muscle angiogenesis [46]. These studies suggest that the increased capillary density found in mammals with high MMRs is indexing higher levels of circulating VEGF. Given the known importance of VEGF for brain growth and neurogenesis, this link suggests that circulating neurotrophins may be playing a role in the association between brain size and MMR described above.

Thus, previous experimental studies show that variation in exercise capacity is correlated with variation in neurotrophins and growth factors important for brain growth and development. It is possible that increases in circulating neurotrophins and growth factors are both present during growth and development. However, the hypothesized mechanism detailed above requires more careful testing. New data on comparative baseline levels of BDNF, IGF-1 and VEGF among athletic and non-athletic species may resolve aspects of the evolution of increased brain size in some mammalian groups. Selection experiments that include specific studies of brain growth and development in mammals that evolved to run long distances may help us understand how exercise and brain size interact across taxa and across evolutionary time.

Finally, it is important to recognize that evolutionary changes in brain size and exercise need not represent an adaptive response between the two, but rather may reflect independent selection on exercise, brain size, or both. For example, selection may have acted on circulating levels of VEGF and IGF-1 for their ability to improve exercise performance (tissue repair, angiogenesis, and glucose metabolism [3], [36], [37]). In this case, the evolutionary increase in these circulating compounds could lead to a change in brain size, without selection acting on brain size itself.

### Conclusions

In this study, we have shown an association between total brain size and the capacity for aerobically supported, endurance-type exercise across a wide range of mammals. This correlation led to the development of a novel hypothesis that details a possible evolutionary mechanism. The evolution of exercise capacity may lead to the upregulation of neurotrophins and growth factors that increase brain growth and development. This explanation is grounded in experimental data showing that brain size is positively correlated with exercise capacity in rodent models and the evolution of increased voluntary wheel running in rodents is associated with increased circulating neurotrophins and growth factors. Additionally, data relating MMR and capillary volume in skeletal muscles suggest that taxa with relatively high MMRs also have high levels of VEGF to maintain those networks. Although it is unlikely that this mechanism would explain a large amount of brain size differences, our results suggest that exercise accounts for approximately 20% of brain size variation in this sample. Thus, a simple mechanism such as the one detailed above may very well explain this modest amount of brain size variation.

Although this study shows that exercise capacity and brain size are connected among mammals, there are exceptions to this relationship that suggest there are many selection pressures that lead to brain size variation [48]. First, when our sample is reduced to wild-only taxa, the correlation between raw variables remains significant, however the PIC analysis is not (p_raw_ = 0.048, p_PIC_ = 0.069). We believe these results reflect a lack of statistical power due to a small sample size in the wild-only analyses, rather than reflecting an actual difference in results. A larger sample size is required to better understand whether there are actual differences in how exercise and neurobiology interact in wild and domesticated taxa. Second, humans do not have greatly increased MMRs, yet have brains much larger than other mammals. Human brain size, and indeed portions of brain size not explained by MMR in other mammals, likely evolved due to changes in diet, social organization, and foraging complexity [17], [49]–[51]. However, our results do not rule out a possible role for exercise in human brain size evolution compared to our closest living relatives. Humans are considered to be exceptional endurance athletes compared to other primates [52], and may therefore have higher MMRs than other primate taxa. It is possible that an increase in exercise capacity during human evolution [52], [53] played a role in the evolution of the human brain, however data on nonhuman primate MMRs required to test this hypothesis do not currently exist.

Thus, our results support a broader relationship between exercise and neurobiology than has been previously shown in intra-specific experimental work. Despite the human exception, it is clear that across nonhuman mammals, increased exercise capacity is associated with increased brain size. This result suggests the intriguing possibility that a portion of brain size variation in nonhuman mammals is not related to selection for cognitive capacity, but rather is due to differences in aerobic activity. Finally, our results lay the foundation for future experimental work examining the possible evolutionary mechanisms behind this broad relationship between exercise and neurobiology. It is possible that selection for increased exercise capacity led to increased circulating levels of IGF-1, VEGF, and BDNF. This hypothesized evolutionary mechanism requires further testing, but if true, may help explain why exercise and brain size are associated across time and taxon.
